# NIDA’s medication development priorities in response to the Opioid Crisis: ten most wanted

**DOI:** 10.1038/s41386-018-0292-5

**Published:** 2018-12-07

**Authors:** Kurt Rasmussen, David A. White, Jane B. Acri

**Affiliations:** Division of Therapeutics and Medical Consequences, National Institute on Drug Abuse, 6001 Executive Blvd, Bethesda, MD 20892 USA

**Keywords:** Pharmacology, Addiction

The United States is in the midst of a horrific problem. The rampant misuse of opioid drugs (both prescribed and illegal), now known as the Opioid Crisis, has had grave effects on both the public health and the well-being of our society. In 1 year, 2017, it is estimated that almost as many Americans died from opioid-related overdose as died in the entire Vietnam War [1]. In response to the problem, the White House has declared the Opioid Crisis a national Public Health Emergency under federal law [2].

The causes of the Opioid Crisis are complex and multifaceted and a solution will require a Herculean, integrated effort from disparate components of society. Changes in both the public and private sectors (e.g., revisions in: health care policy; medical education; business regulation; deployment of existing medications; local and state justice systems) will be needed to address this crisis. In an effort to leverage science to help address the problem, the National Institutes of Health (NIH) has launched the HEAL (Helping to End Addiction Long-term) Initiative, an aggressive, trans-agency effort to speed scientific solutions to the Opioid Crisis [3]. This initiative will nearly double funding for research on opioid misuse/addiction and pain. As part of the NIH, the National Institute on Drug Abuse (NIDA) is devoted to addressing this crisis in multiple ways. NIDA will coordinate four overarching research projects around the country: the Focused Opioid Use Disorder (OUD) Medications Development Research Project; the HEALing Communities Study; the Clinical Trials Network OUD Research Enhancement Project; and the Justice Community Opioid Innovation Network [4]. The medication development component of this four-pronged effort includes aiding the development of novel pharmacotherapies, behavioral therapies and devices for the treatment of opioid overdose and OUD.

Our science can have political, economic and social ramifications. Indeed, the introduction of safe and effective therapeutics, while unlikely to be a panacea, has the potential to transform not only health outcomes for individual patients, but anachronistic societal attitudes towards diseases, especially brain diseases. In this regard, we hope that the introduction of new safe and effective medications for OUD will enlighten the public discourse around opioid addiction and those suffering from it. In an effort to specifically speed the development of pharmacotherapies for the treatment of OUD and reach NIDA’s stated goal of 15 Investigational New Drugs (INDs) and 5 New Drug Applications (NDAs) submitted to the Food and Drug Administration (FDA), NIDA’s Division of Therapeutics and Medical Consequences (DTMC) has created a list of medication development priorities.

The mechanisms listed in Table 1 are NIDA’s DTMC highest priority pharmacological targets for the development of novel therapeutics to treat opioid overdose and OUD *in the near term*. The list does not include mechanisms of existing OUD medications and the mechanisms are listed in no particular order. While the existing medications (e.g., buprenorphine, methadone, naloxone, naltrexone, lofexidine) have demonstrable utility in the treatment of OUD, they are not without limitations. Indeed, problematic residual symptoms and discontinuation rates plague these treatments [5, 6], leaving a deceptively cavernous un-met medical need that could be addressed, at least in part, by new medications.

**Table 1:** NIDA's DTMC ten most wanted pharmacological mechanisms for the rapid development of therapeutics in response to the Opioid Crisis

NIDA’s DTMC ten most wanted
Orexin-1 or 1/2 antagonists or NAMs [17–19]
Kappa opioid antagonists or NAMs [20, 21]
GABA-B agonists or PAMs [22, 23]
Muscarinic M5 antagonists or NAMs [24, 25]
AMPA antagonists, NAMs or PAMs [26–28]
NOP/ORL agonists, antagonists, NAMs or PAMs [29–31]
mGluR2/3 agonists or PAMs [32–34]
Ghrelin antagonists or NAMs [35, 36]
Dopamine D3 partial agonists, PAMs, antagonists or NAMs [37, 38]
Cannabinoid CB-1 antagonists or NAMs [39, 40]

*PAM* positive allosteric modulator, *NAM* negative allosteric modulator, *AMPA* α-amino-3-hydroxy-5-methyl-4-isoxazolepropionic acid, *GABA* γ-aminobutyric acid, *NOP* nociceptin opioid peptide receptor, *ORL* opioid receptor like, *mGluR* metabotropic glutamate receptor, *5HT* 5-hydroxytryptamine, *MOP* mu opioid protein

Other mechanisms of interest:

5HT2C agonists or PAMs, with or without 5HT2A antagonist/NAM activity [41, 42]

Biased Mu Opioid agonists or PAMs [43, 44]

NOP/MOP bifunctional agonists or PAMs [45, 46]

Respiratory stimulants (including nicotinic agonists) [47, 48]

Our goal is to help deliver new treatment options to the millions of patients and physicians battling OUD. At this point in time, we feel compounds with the mechanisms-of-action listed in Table 1 have the highest probability of a path to FDA approval for the treatment of some aspect of OUD *in the near term*. An important component of this list are allosteric modulators. Based on their suppression/augmentation of endogenous responses, negative allosteric modulators (NAMs) and positive allosteric modulators (PAMs) may provide more physiologically relevant effects compared with agonists and antagonists acting on the same receptor, which may ultimately result in improved clinical outcomes [7]. It is important to note that due to the complexity of the addiction cycle, different stages of the disease (e.g., transition from sporadic to chronic use, acute withdrawal, delayed relapse) are likely to have different (albeit overlapping) pathophysiologies [8]. Thus, there is unlikely to be a “silver bullet” among these mechanisms for the treatment of OUD and medications with these mechanisms-of-action are likely to be useful at different stages of the addiction cycle. In addition, it is important to remember, as has been clearly demonstrated from the treatment of major depressive disorder [9], pharmacotherapies can have greater impact when paired with effective psychosocial interventions. Indeed, two key components of NIDA’s treatment development efforts are the development of novel behavioural and device treatments. Ultimately, we anticipate multiple medications, integrated with both psychosocial interventions and potentially devices, employed in an orchestrated fashion, will be needed to achieve truly effective treatments “tailored” for maximal efficacy in different individuals.

We have determined our “most wanted” mechanisms based on data from published literature and internal studies that we feel have the most direct relevance to desirable treatment effects and clinical endpoints for OUD. Importantly, most of these mechanisms are active in more than one model, and for more than one drug of abuse, which presents the intriguing possibility of their potential efficacy in treating polydrug abuse or other substance use disorders. As with all brain diseases, the translatability and predictive validity of data from preclinical assays for clinical efficacy have been disputed [10–12], but see ref. [13]. As such, for each mechanism-of-action, we invite: (1) critical preclinical data that will either strengthen, sink or revise the hypothesis; (2) translational data which would help define the predictive validity of the preclinical data; and (3) clinical trials or laboratory studies that will definitively test the hypothesis in humans.

We do not propose this list as a static be-all and end-all; but, rather, a dynamic clarion call that will evolve with advances in the field. Indeed, we hope that proposing this list will help spur the future research that will ultimately antiquate it. The list is biased towards *proximal action* as, quite literally, people are dying. In this regard, mechanisms that have compounds already in, or close to, clinical development and have a high probability of a path to FDA approval have been emphasized. As these mechanisms are tested in the clinic, they will move off the list either as new therapeutics for the millions of patients battling OUD or as “learning opportunities” (as there is much to be learned even from clinical trial “failures”). Of course, there is robust, on-going research into uncovering additional mechanisms, not yet on the list, that could be useful for the treatment of OUD. Examples of entirely new directions for novel OUD treatments include epigenetic [14], micro RNA [15] and neuroimmune targets [16]. We anticipate, and fervently hope, additional research will help new mechanisms “percolate up” to warrant inclusion on this list. Additionally, we hope to engender vigorous scientific debate on what should, and should not, be on this list. We welcome your feedback, encourage suggestions for additional novel mechanisms of action, and, as always, invite more data!

## References

[1] National Institute on Drug Abuse. Overdose Death Rates. 2018; https://www.drugabuse.gov/related-topics/trends-statistics/overdose-death-rates. Accessed 20 November 2018.

[2] The White House. The Opioid Crisis. 2017; https://www.whitehouse.gov/opioids/. Accessed 20 November 2018.

[3] National Institutes of Health. NIH launches HEAL Initiative, doubles funding to accelerate scientific solutions to stem national opioid epidemic. 2018; https://www.nih.gov/news-events/news-releases/nih-launches-heal-initiative-doubles-funding-accelerate-scientific-solutions-stem-national-opioid-epidemic. Accessed 20 November 2018.

[4] National Institute on Drug Abuse. NIDA’s role in the NIH HEAL Initiative. 2018; https://www.drugabuse.gov/drugs-abuse/opioids/nidas-role-in-nih-heal-initiative. Accessed 20 November 2018.

[5] Koustova E, McCaffrey A, Prikhodko VG, Sazonova IY, Volkow ND. Research to help combat opioid crisis: using patients’ and families’ need assessment to inform research agenda in substance use disorders. Sci Trans Res. 2018; submitted.

[6] Morgan JR, Schackman BR, Leff JA, Linas BP, Walley AY (2018). Injectable naltrexone, oral naltrexone, and buprenorphine utilization and discontinuation among individuals treated for opioid use disorder in a United States commercially insured population. J Subst Abus Treat.

[7] Conn PJ, Christopoulos A, Lindsley CW (2009). Allosteric modulators of GPCRs: a novel approach for the treatment of CNS disorders. Nat Rev Drug Disc.

[8] Kwako LE, Momenan R, Litten RZ, Koob GF, Goldman D (2016). Addictions neuroclinical assessment: a neuroscience-based framework for addictive disorders. Biol Psychiatry.

[9] Ijaz S, Davies P, Williams CJ, Kessler D, Lewis G, Wiles N (2018). Psychological therapies for treatment-resistant depression in adults. Cochrane Database Syst Rev.

[10] Katz JL, Higgins ST (2003). The validity of the reinstatement model of craving and relapse to drug use. Psychopharmacol (Berl).

[11] Pierce RC, O’Brien CP, Kenny PJ, Vanderschuren LJ (2012). Rational development of addiction pharmacotherapies: successes, failures, and prospects. Cold Spring Harb Perspect Med.

[12] Reiner DJ, Fredriksson I, Lofaro OM, Bossert JM, Shaham Y. Relapse to opioid seeking in rat models: behavior, pharmacology and circuits. Neuropsychopharmacology. Published online 6 October 2018. 10.1038/s41386-018-0234-2. 10.1038/s41386-018-0234-2 PMC6333846 30293087

[13] Spanagel R (2017). Animal models of addiction. Dialog- Clin Neurosci.

[14] Walker DM, Nestler EJ (2018). Neuroepigenetics and addiction. Handb Clin Neurol.

[15] Smith ACW, Kenny PJ (2018). MicroRNAs regulate synaptic plasticity underlying drug addiction. Genes Brain Behav.

[16] Lacagnina MJ, Rivera PD, Bilbo SD (2017). Glial and neuroimmune mechanisms as critical modulators of drug use and abuse. Neuropsychopharmacology.

[17] Baimel C, Bartlett SE, Chiou LC, Lawrence AJ, Muschamp JW, Patkar O (2015). Orexin/hypocretin role in reward: implications for opioid and other addictions. Br J Pharmacol.

[18] Alizamini MM, Farzinpour Z, Ezzatpanah S, Haghparast A (2017). Role of intra-accumbal orexin receptors in the acquisition of morphine-induced conditioned place preference in the rats. Neurosci Lett.

[19] Sahafzadeh M, Karimi-Haghighi S, Mousavi Z, Haghparast A (2018). Role of the orexin receptors within the nucleus accumbens in the drug priming-induced reinstatement of morphine seeking in the food deprived rats. Brain Res Bull.

[20] Robinson SE (2006). Buprenorphine-containing treatments: place in the management of opioid addiction. CNS Drugs.

[21] Carlezon WA, Krystal AD (2016). Kappa-opioid antagonists for psychiatric disorders: from bench to clinical trials. Depress Anxiety.

[22] Xi ZX, Stein EA (1999). Baclofen inhibits heroin self-administration behavior and mesolimbic dopamine release. J Pharmacol Exp Ther.

[23] Spano MS, Fattore L, Fratta W, Fadda P (2007). The GABAB receptor agonist baclofen prevents heroin-induced reinstatement of heroin-seeking behavior in rats. Neuropharmacology.

[24] Basile AS, Fedorova I, Zapata A, Liu X, Shippenberg T, Duttaroy A (2002). Deletion of the M5 muscarinic acetylcholine receptor attenuates morphine reinforcement and withdrawal but not morphine analgesia. Proc Natl Acad Sci Usa.

[25] Raffa RB (2009). The M5 muscarinic receptor as possible target for treatment of drug abuse. J Clin Pharm Ther.

[26] Rasmussen K, Kendrick WT, Kogan JH, Aghajanian GK (1996). A selective AMPA antagonist, LY293558, suppresses morphine withdrawal-induced activation of locus coeruleus neurons and behavioral signs of morphine withdrawal. Neuropsychopharmacology.

[27] Haw AJ, Meyer LC, Greer JJ, Fuller A (2016). Ampakine CX1942 attenuates opioid-induced respiratory depression and corrects the hypoxaemic effects of etorphine in immobilized goats (Capra hircus). Vet Anaesth Analg.

[28] Siahposht-Khachaki A, Fatahi Z, Yans A, Khodagholi F, Haghparast A (2017). Involvement of AMPA/Kainate glutamate receptor in the extinction and reinstatement of morphine-Induced conditioned place preference: a behavioral and molecular study. Cell Mol Neurobiol.

[29] Rutten K, De VJ, Bruckmann W, Tzschentke TM (2010). Effects of the NOP receptor agonist Ro65-6570 on the acquisition of opiate- and psychostimulant-induced conditioned place preference in rats. Eur J Pharmacol.

[30] Lin AP, Ko MC (2013). The therapeutic potential of nociceptin/orphanin FQ receptor agonists as analgesics without abuse liability. ACS Chem Neurosci.

[31] Zaveri NT (2016). Nociceptin opioid receptor (NOP) as a therapeutic target: progress in translation from preclinical research to clinical utility. J Med Chem.

[32] Vandergriff J, Rasmussen K (1999). The selective mGlu2/3 receptor agonist LY354740 attenuates morphine-withdrawal-induced activation of locus coeruleus neurons and behavioral signs of morphine withdrawal. Neuropharmacology.

[33] Popik P, Kozela E, Pilc A (2000). Selective agonist of group II glutamate metabotropic receptors, LY354740, inhibits tolerance to analgesic effects of morphine in mice. Br J Pharmacol.

[34] Bossert JM, Gray SM, Lu L, Shaham Y (2006). Activation of group II metabotropic glutamate receptors in the nucleus accumbens shell attenuates context-induced relapse to heroin seeking. Neuropsychopharmacology.

[35] Maric T, Sedki F, Ronfard B, Chafetz D, Shalev U (2012). A limited role for ghrelin in heroin self-administration and food deprivation-induced reinstatement of heroin seeking in rats. Addict Biol.

[36] Engel JA, Nylander I, Jerlhag E (2015). A ghrelin receptor (GHS-R1A) antagonist attenuates the rewarding properties of morphine and increases opioid peptide levels in reward areas in mice. Eur Neuropsychopharmacol.

[37] Galaj E, Manuszak M, Babic S, Ananthan S, Ranaldi R (2015). The selective dopamine D3 receptor antagonist, SR 21502, reduces cue-induced reinstatement of heroin seeking and heroin conditioned place preference in rats. Drug Alcohol Depend.

[38] You ZB, Gao JT, Bi GH, He Y, Boateng C, Cao J (2017). The novel dopamine D3 receptor antagonists/partial agonists CAB2-015 and BAK4-54 inhibit oxycodone-taking and oxycodone-seeking behavior in rats. Neuropharmacology.

[39] Caille S, Parsons LH (2006). Cannabinoid modulation of opiate reinforcement through the ventral striatopallidal pathway. Neuropsychopharmacology.

[40] He XH, Jordan CJ, Vemuri K, Bi GH, Zhan J, Gardner EL (2018). Cannabinoid CB1 receptor neutral antagonist AM4113 inhibits heroin self-administration without depressive side effects in rats. Acta Pharmacol Sin.

[41] Neelakantan H, Holliday ED, Fox RG, Stutz SJ, Comer SD, Haney M (2017). Lorcaserin suppresses oxycodone self-administration and relapse vulnerability in rats. ACS Chem Neurosci.

[42] Kohut SJ, Bergman J (2018). Lorcaserin decreases the reinforcing effects of heroin, but not food, in rhesus monkeys. Eur J Pharmacol.

[43] Crowley RS, Riley AP, Sherwood AM, Groer CE, Shivaperumal N, Biscaia M (2016). Synthetic studies of neoclerodane diterpenes from Salvia divinorum: identification of a potent and centrally acting mu opioid analgesic with reduced abuse liability. J Med Chem.

[44] Manglik A, Lin H, Aryal DK, McCorvy JD, Dengler D, Corder G (2016). Structure-based discovery of opioid analgesics with reduced side effects. Nature.

[45] Toll L, Khroyan TV, Polgar WE, Jiang F, Olsen C, Zaveri NT (2009). Comparison of the antinociceptive and antirewarding profiles of novel bifunctional nociceptin receptor/mu-opioid receptor ligands: implications for therapeutic applications. J Pharmacol Exp Ther.

[46] Ding H, Kiguchi N, Yasuda D, Daga PR, Polgar WE, Lu JJ (2018). A bifunctional nociceptin and mu opioid receptor agonist is analgesic without opioid side effects in nonhuman primates. Sci Transl Med.

[47] Naeije R, Lejeune P, Vachiery JL, Leeman M, Melot C, Hallemans R (1990). Restored hypoxic pulmonary vasoconstriction by peripheral chemoreceptor agonists in dogs. Am Rev Respir Dis.

[48] Dehkordi O, Haxhiu MA, Millis RM, Dennis GC, Kc P, Jafri A (2004). Expression of alpha-7 nAChRs on spinal cord-brainstem neurons controlling inspiratory drive to the diaphragm. Respir Physiol Neurobiol.

